# Monoclonal Antibodies to S and N SARS-CoV-2 Proteins as Probes to Assess Structural and Antigenic Properties of Coronaviruses

**DOI:** 10.3390/v13101899

**Published:** 2021-09-22

**Authors:** Rinki Kumar, Neil D. Christensen, Rebecca J. Kaddis Maldonado, Maria C. Bewley, Alexandria Ostman, Malgorzata Sudol, Eunice C. Chen, Natalie W. Buchkovich, Abhinay Gontu, Meera Surendran Nair, Ruth H. Nissly, Allen M. Minns, Vivek Kapur, Randall Rossi, Suresh V. Kuchipudi, Scott E. Lindner, Leslie J. Parent, John M. Flanagan, Nicholas J. Buchkovich

**Affiliations:** 1 Department of Microbiology & Immunology, Penn State College of Medicine, Hershey, PA 17033, USA; rkumar1@pennstatehealth.psu.edu (R.K.); aostman@pennstatehealth.psu.edu (A.O.); nbuchkovich1@pennstatehealth.psu.edu (N.W.B.); lparent@psu.edu (L.J.P.); 2 Department of Pathology, Penn State College of Medicine, Hershey, PA 17033, USA; 3 Huck Institutes of the Life Sciences, Penn State University, University Park, PA 16802, USA; rah38@psu.edu (R.H.N.); amm504@psu.edu (A.M.M.); vkapur@psu.edu (V.K.); rmr29@psu.edu (R.R.); skuchipudi@psu.edu (S.V.K.); scott.lindner@psu.edu (S.E.L.); jflanagan@psu.edu (J.M.F.); 4 Department of Medicine, Penn State College of Medicine, Hershey, PA 17033, USA; rkaddis@pennstatehealth.psu.edu (R.J.K.M.); msudol@pennstatehealth.psu.edu (M.S.); echen@pennstatehealth.psu.edu (E.C.C.); 5 Department of Biochemistry & Molecular Biology, Penn State College of Medicine, Hershey, PA 17033, USA; mcb21@psu.edu; 6 Department of Veterinary and Biomedical Sciences, Penn State University, University Park, PA 16802, USA; abhinay@psu.edu (A.G.); mms7306@psu.edu (M.S.N.); 7 Department of Animal Science, Penn State University, University Park, PA 16802, USA; 8 Department of Biochemistry and Molecular Biology, Penn State University, University Park, PA 16802, USA

**Keywords:** COVID-19, SARS-CoV-2, coronavirus, antibodies, spike protein, nucleocapsid protein

## Abstract

Antibodies targeting the spike (S) and nucleocapsid (N) proteins of severe acute respiratory syndrome coronavirus 2 (SARS-CoV-2) are essential tools. In addition to important roles in the treatment and diagnosis of infection, the availability of high-quality specific antibodies for the S and N proteins is essential to facilitate basic research of virus replication and in the characterization of mutations responsible for variants of concern. We have developed panels of mouse and rabbit monoclonal antibodies (mAbs) to the SARS-CoV-2 spike receptor-binding domain (S-RBD) and N protein for functional and antigenic analyses. The mAbs to the S-RBD were tested for neutralization of native SARS-CoV-2, with several exhibiting neutralizing activity. The panels of mAbs to the N protein were assessed for cross-reactivity with the SARS-CoV and Middle East respiratory syndrome (MERS)-CoV N proteins and could be subdivided into sets that showed unique specificity for SARS-CoV-2 N protein, cross-reactivity between SARS-CoV-2 and SARS-CoV N proteins only, or cross-reactivity to all three coronavirus N proteins tested. Partial mapping of N-reactive mAbs were conducted using truncated fragments of the SARS-CoV-2 N protein and revealed near complete coverage of the N protein. Collectively, these sets of mouse and rabbit monoclonal antibodies can be used to examine structure/function studies for N proteins and to define the surface location of virus neutralizing epitopes on the RBD of the S protein.

## 1. Introduction

Coronaviruses, such as SARS-CoV, MERS-CoV, and SARS-CoV-2, are zoonotic infections that have been transmitted from animal hosts into the human population in the past two decades with devastating (and most recently) global effects on human health, welfare, mobility, and behavior [1,2]. The current coronavirus disease-2019 (COVID-19) pandemic caused by SARS-CoV-2 continues its global spread. Emerging new variants, such as the delta and lambda variants, are adding to these concerns and creating new challenges to controlling the pandemic [3]. While COVID-19 vaccines were quickly generated and mass vaccination drives are underway throughout the world [4], a constant threat looms due to the rapidly evolving nature of the virus and the emergence of variants of concern [5,6].

A limited understanding of the molecular details underlying the SARS-CoV-2 proteins poses a challenge in identifying candidate therapies. To counteract SARS-CoV-2 infection and the associated COVID-19 pathology, it is crucial to understand how this virus hijacks the host during infection and to apply this knowledge to identify novel therapeutic approaches [7]. Furthermore, it is crucial to have rapid and accurate diagnostic tools to detect SARS-CoV-2 at the early stages of the disease to ensure a better treatment outcome and to reduce transmission. To facilitate this development, key reagents such as high-quality antibodies to the viral proteins are indispensable. These reagents can be useful for the analysis of structural and antigenic features of the coronavirus virions, which will be essential to better understand the impact of vaccines on current infections, on mutations that lead to vaccine escape, and for functional analyses of viral proteins in the replication cycle of coronaviruses. The spike (S) protein binding to the ACE2 receptor is key for viral infection via attachment and entry into target cells [8,9]. While antibodies targeting the spike protein of SARS-CoV-2 present a promising approach to combat the COVID-19 pandemic, the utility of antibodies against other viral proteins should not be overlooked. Notably, antibodies to the nucleocapsid (N) protein of SARS-CoV-2 are more sensitive than the spike protein antibody for detecting early infection [10].

In this study, we generate panels of monoclonal antibodies to the S and N structural proteins of SARS-CoV-2 to provide sets of unique probes and assessed their functional activities (virus neutralization), antigenicity, and epitope specificity. Both mouse and rabbit monoclonal antibodies (mAbs) to the S protein were developed and neutralizing activity was observed from both sources. Anti-N mAbs were analyzed for specificity using N proteins from SARS-CoV, SARS-CoV-2, and MERS-CoV, as well as fragments of the SARS-CoV-2 N protein. Despite the high level of conservation between the N proteins of these three coronaviruses, we were able to identify several SARS-CoV-2 type-specific N-reactive mAbs, and these may provide highly useful specificity controls for various immunoassays.

## 2. Materials and Methods

### 2.1. Plasmids, Protein Expression and Purification

Gibson assembly [11] was utilized to clone the N proteins of various coronaviruses into a pET28 expression vector for bacterial expression in the *Escherichia coli* BL-21(DE3)RIL host. First, pET28 (fragment 1) was digested with NdeI and AgeI. PCR products with overlapping ends (fragment 2) were generated with the following primer sets: SARS-CoV N forward primer 5′-AATTTTGTTTAACTTTAAGAAGGAGATATACCAT ATGTCTGATAATGGACCCCAATCAAACCAACGT- 3′ and reverse primer 5′-CCATTG TCATCGCTAAACCGGTTTATTATTATGCCTGAGTTGAATCAGCAGAAGC-3′; SARS-CoV-2 N forward primer 5′-AATTTTGTTTAACTTTAAGAAGGAGATATACCATATGT CTGATAATGGACCCCAAAATCAGCGAAATGCACCCCGCATTA-3′ and reverse primer 5′-CCATTGTCATCGCTAAACCGGTTTATTATTAGGCCTGAGTTGAGTCAGCAC TGCTCATGGATTG-3′; SARS-CoV-2 N 1–174 forward primer 5′-AATTTTGTTTAACTTT AAGAAGGAGATATACCATATGTCTGATAATGGACCCCAAAATCAGCGAAATGCACCCCGCATTA-3′ and reverse primer 5′-CCATTGTCATCGCTAAACCGGTTTATTATTATTCTGCGTAGAAGCCTTTTGGCAATGT-3′; SARS-CoV-2 N 41–174 forward primer 5′-AATTTTGTTTAACTTTAAGAAGGAGATATACCATATGCGGCCCCAAGGTTT ACCCAATAATACT-3′ and reverse primer 5′-CCATTGTCATCGCTAAACCGGTTTATT ATTATTCTGCGTAGAAGCCTTTTGGCAATGT-3′; SARS-CoV-2 N 247–364 forward primer 5′-AATTTTGTTTAACTTTAAGAAGGAGATATACCATATGACTAAGAAATCTGCTGCTGAGGCTTCTAAGAAG-3′ and reverse primer 5′-CCATTGTCATCGCTAAACCGGTTTATTATTATGGGAATGTTTTGTATGCGTCAATATGCTTATT-3′; MERS-CoV N forward primer 5′-AATTTTGTTTAACTTTAAGAAGGAGATATACCATATGGCAT CCCCTGCTGCACCT-3′ and reverse primer 5′-CCATTGTCATCGCTAAACCGGTTTATTATTAATCAGTGTTAACATCAATCATTGGACCAGG-3′. Three stop codons were cloned after each gene to prevent the translation of a His tag. Gene blocks of the SARS-CoV N sequence from GenBank AY291451.1 (Genewiz, South Plainfield, NJ, USA) and the MERS-CoV N sequence from GenBank NC_038294.1 (Integrated DNA technologies) served as templates. The source of the SARS-CoV-2 N protein open reading frame was the N protein positive control vector (Integrated DNA Technologies, Coralville, IA, USA). For the mammalian expression of N protein in 293T cells, the SARS-CoV-2 N protein open reading frame (ORF) was amplified from an N protein positive control vector (IDT), with XbaI/NotI sites added during amplification (GACTTCTAGAATGTCTGATAATGGACCC and GTACGCGGCCGCTTAGGCCTGAGTTGAGTC) and subsequently inserted into a mammalian expression vector using NheI/NotI restriction sites. The SARS-CoV N protein expression plasmid was similarly cloned following ORF amplification from the pET28a-CoV-N plasmid described above.

The following reagents were produced under HHSN272201400008C and obtained through BEI Resources, NIAID, NIH: Vector pCAGGS Containing the SARS-Related Coronavirus 2, Wuhan-Hu-1 Spike Glycoprotein Gene, NR-52310 and Vector pCAGGS Containing the SARS-Related Coronavirus 2, Wuhan-Hu-1 Spike Glycoprotein Receptor Binding Domain (S-RBD), NR-52309. The S-RBD was purified from 293T cells according to published protocols [12,13].

For large-scale protein expression in *E. coli* and subsequent protein purification, a saturated MDG starter culture was diluted from 1:250 to 1:1000 fold into 250 mL of ZYM5052 media supplemented with 100 μg/mL kanamycin and 25 μg/mL chloramphenicol [14]. The cultures were initially grown for 3–4 h at 37 °C (O.D. 600 < 1) and then transferred to 16 °C (full-length coronavirus N constructs and SARS-CoV-2 N Δ(2–49)) or 24 °C (all remaining constructs) and grown until they reached saturation (typically 14–18 h) as judged by the absence of an increase in O.D. 600 nm between readings taken ~1hr apart. The cultures were then harvested by centrifugation for 10 min at 6000× *g*, the supernatant decanted, and the cell pellets frozen and stored at −20 °C until used.

Cells (3–4 gm wet weight) containing overexpressed coronavirus N constructs were resuspended in 50 mL of lysis buffer (25 mM Tris·HCl pH 8.0; 500 mM NaCl; 0.1 mM EDTA and 1 tablet of Roche complete protease inhibitors) and lysed by three passes through a microfluidizer (MP110P, Microfluidics, Westwood, MA, USA) at 13,000 psi. The cell lysate was then cleared by centrifugation at 25,000× *g* and 4 °C for 30 min and the resulting supernatant was adjusted to 0.14% *v*/*v* polyethylenimine-P (PEI) by addition from a 10% stock (pH 7.8). The PEI precipitated debris was removed by centrifugation at 18,000× *g* and 4 °C for 10 min and the supernatant was transferred to a beaker in an ice water bath. The N protein was precipitated from this solution by the addition of solid ammonium sulfate to a final concentration of 80% (*w*/*v*). This solution was stirred for 15–30 min on ice and the precipitate was collected by centrifugation at 21,000× *g* for 30 min. The supernatant was then carefully decanted, and the pellets dried by inversion for 5 min at room temperature. Any residual supernatant was removed from the rim of the centrifuge tubes with a Kimwipe (Kimberly-Clark).

For full-length coronavirus and SARS-CoV-2 N Δ(2–49) constructs the drained Am_2_SO_4_ pellets were resuspended completely in 50 mL of ice-cold resuspension buffer (25 mM Tris·HCl pH 8.0, 50 mM NaCl; 0.1 mM EDTA; 10% *v*/*v* glycerol and 0.1× protease inhibitors (ProBlock, Gold Biochem, St. Louis, MO, USA ) and the suspension was centrifuged at 20,000× *g* at 4 °C for 30 min. The supernatants were loaded onto a 10 mL Poros HS-50 (Thermo Fisher, Waltham, MA, USA) column equilibrated in 25 mM Tris·HCl pH 8.0, 100 mM NaCl; 0.1 mM EDTA, and 10% *v*/*v* glycerol, and the unbound and weakly bound material was removed by washing the column with 5 column volumes (CV) of loading buffer. The bound protein was eluted in a 10 CV linear salt gradient from 0.1–1.5 M NaCl in an equilibration buffer. The peak N-containing fractions were identified by Coomassie-stained SDS-PAGE analysis pooled and concentrated in a 15 mL amicon-30 spin concentrator to an apparent concentration of 10–15 mg/mL based upon A280 values using extinction coefficients determined in ExPasy Protparam [15]. The proteins were further purified by size exclusion chromatography on a Superdex S200 column equilibrated in 20 mM HEPES pH 7.5; 300 mM NaCl; and 0.1 mM EDTA. Peak fractions (Coomassie-stained SDS-PAGE) were concentrated to 8–15 mg/mL using the appropriate extinction 280 nm constant, aliquoted, snap-frozen in liquid N_2_, and stored at either −20 °C or −80 °C.

For the remaining constructs, the Am_2_SO_4_, pellets were resuspended in 20 mL of resuspension buffer and dialyzed overnight against the same buffer to ensure that the ionic strength was <100 mM before loading onto the Poros HS-50 column. These constructs were purified by a similar procedure to the full-length ones with the exception that they were eluted from the ion exchange column in a linear gradient from 0.075–1 M NaCl, and for most constructs with expected molecular weights > 40 kDa, 10 kDa cutoff Amicon separators were used.

### 2.2. Immunization of Mice and Rabbits—Preparation of Sera and Mabs

All animal studies were reviewed and approved by the Penn State University College of Medicine IACUC (PROTO201900719). Animal studies followed guidelines from the NIH regarding the care and use of animals in research. Rabbits and mice were immunized by standard methods using purified viral protein admixed with Sigma adjuvant (RIBI adjuvant) at a 1:1 ratio as described by the manufacturer. Animals were immunized with 100–200 µg of protein in a volume of 100 µL of adjuvanted preparation by i.p. immunization of mice and 200 µL for i.m. immunization of rabbits. Booster immunizations (2 per animal) were conducted 2–3 weeks after each immunization with a final booster in saline. Animals were anesthetized prior to the collection of blood samples and euthanized for terminal harvesting of tissues and blood.

Monoclonal antibodies were prepared by standard hybridoma fusion technology with polyethylene glycol as previously described [16]. The fusion partner for mouse hybridomas was P3XAg.8.683 (ATCC), and for rabbits was line 240E from Katherine Knight [17]. The latter line was sub-cloned several times to develop a more stable fusion partner. Rabbit hybridomas were prepared from rabbit spleen cells previously preserved in DMSO and stored in liquid nitrogen tanks. The spleen cells were thawed, washed, and fused with line 240E using the same procedure as for the mouse hybridomas. Fusion reactions were plated into 96-well flat-bottomed cell culture plates and hybridomas were selected using standard hypoxanthine/aminopterin/thymidine selection methods. Growing hybridomas were marked on the wells of the plates and the supernatants of these wells were tested in ELISA for reactivity to viral proteins as described below. Positive wells were selected for cloning and several hybridomas were adapted to serum-free media (Thermo Fisher) and high-titer antibody preparations were prepared using CELLine culture vessels (Wheaton, Staffordshire, UK).

### 2.3. Enzyme-Linked Immunosorbent Assays (ELISA)

ELISAs were conducted using standard procedures [18]. Culture supernatants from wells containing hybridomas were initially screened for reactivity to intact protein attached to ELISA plate wells using alkaline buffer [19]. Positive cultures were expanded, cloned and the clones retested for reactivity, expanded again, and stored as aliquots for various assays (ELISA, Western blotting, IF, and neutralization). Dilutions of culture supernatant were titrated in the ELISA to establish ½ maximum binding activity (dilution of supernatant that produced 50% of maximum O.D. values). Selected hybridomas were conditioned to grow in serum-free media (MilliporeSigma, St. Louis, MO, USA) and high-titer antibodies were prepared for additional studies.

Sandwich ELISAs were conducted to assess antibody titers as well as to determine competition for binding to S-RBD and N proteins. For S-RBD assays, selected monoclonal antibodies were captured onto ELISA plate wells using anti-mouse IgM or anti-rabbit antibodies (Southern Biotech, Birmingham, AL, USA) and after washing and blocking steps, viral antigens were added to be captured by the appropriate mAb. The captured antigens were then scanned for binding by the panels of mAbs to determine whether there was competition between the capture and detection mAbs. Loss of signal from the detection antibody was considered an indication that the capture mAb was bound to the same or adjacent epitope, preventing the detection mAb from binding.

Denatured and reduced ELISAs were prepared by adding 2-mercaptoethanol to aliquots of the protein antigen (N protein and S-RBD) in an alkaline buffer followed by boiling for 10 min at 100 °C. The antigen preparation was cooled to room temperature then attached directly to ELISA plate wells followed by overnight incubation at 4 °C. The denatured and reduced antigen was detected by standard direct ELISA as described above.

### 2.4. Western Blotting

Total cell lysates were prepared from 293T cells transfected with plasmids expressing N or S protein by lysing cells in RIPA buffer supplemented with 1 mM phenylmethylsulfonyl fluoride (PMSF), 1 mM aprotinin, 0.2 mM Na_3_VO_4_, and 1 µg/mL leupeptin. Supernatants from transfected cells were centrifuged at 20,000 rpm at 4 °C and resuspended in 1× PBS. RIPA supplemented with protease inhibitors was added. All samples were quantified by Bradford Assay. SDS loading dye with (reducing) or without (non-reducing) beta-mercaptoethanol was added to samples to be probed with N antibody and S-antibody, respectively, and boiled at 95 °C. For media samples, the media was collected 72 h post transfection and spun at 500× *g* for 10 min. The supernatant was collected and centrifuged at 120,000× *g* for 3 h over a 30% sucrose cushion. The pellet was directly resuspended in an SDS loading dye and heated to 95 °C. Samples were subjected to SDS-PAGE and transferred to a nitrocellulose membrane, blocked, and then probed with the respective antibodies in 5% *w*/*v* milk in Tris-buffered saline (0.1% *v*/*v* Tween-20). Blots were developed using SuperSignal West Pico PLUS chemiluminescent substrate (Thermo Scientific, Waltham, MA, USA) and imaged on a ChemiDoc MP Imaging System (Bio-Rad, Hercules, CA, USA). Horseradish peroxidase-conjugated secondary antibodies for Western blotting were purchased from Jackson Laboratories (goat anti-rabbit) and GE Healthcare (sheep anti-mouse).

### 2.5. Immunofluorescence Microscopy and Imaging

Coverslips with 293T cells were transfected with the plasmids expressing N or S proteins using X-Treme Gene HP DNA Transfection Reagent (MilliporeSigma) according to the manufacturer’s instructions. Cells were fixed in 4% *v*/*v* paraformaldehyde for 15 min at room temperature and blocked in PBS containing 10% *v*/*v* human serum, 0.5% *v*/*v* Tween-20, and 5% *w*/*v* glycine; Triton X-100 (0.1% *v*/*v*) was added for permeabilization. Primary and secondary antibodies were diluted in blocking buffer. Alexa Fluor 488-conjugated secondary antibodies (Thermo Scientific) were used as secondary antibodies. Coverslips were mounted with ProLong Diamond antifade mountant with DAPI (Thermo Scientific). Images were taken on a C2+ confocal microscope (Nikon, Minato City, Tokyo, Japan). Images were processed using NIS Elements software.

### 2.6. SARS-CoV-2 Microneutralization Assay (VN)

The ability of monoclonal and polyclonal antibodies (pAbs) to neutralize SARS-CoV-2 host-cell infection was determined with a traditional VN assay using the SARS-CoV-2 strain USA-WA1/2020 (NR-52281-BEI resources), as previously described [20]. All VN experiments were conducted under BSL-3 containment conditions in the Eva J. Pell laboratory for advanced biological research at Penn State. The assay was performed in triplicate, and 8 two-fold serial dilutions of the serum or monoclonal antibody supernatant were assessed. Briefly, 100 tissue culture infective dose 50 (TCID50) units of SARS-CoV-2 were added to two-fold dilutions of serum or supernatant and incubated for 1 h at 37 °C. The virus and antibody mixture was added to Vero E6 cells (ATCC CRL-1586), grown in a 96-well microtiter plate, and incubated for three days, after which the host cells were treated for one hour with crystal violet–formaldehyde stain (0.013% *w*/*v* crystal violet, 2.5% *v*/*v* ethanol, and 10% *v*/*v* formaldehyde in 0.01 M PBS). The endpoint of the microneutralization assay was designated as the highest sample dilution, at which all 3, or 2 of 3 wells are not protected from virus infection, as assessed by visual examination.

## 3. Results

### 3.1. Generation of S-RBD Antibodies with Diverse Binding and Neutralization Properties

A set of mouse and rabbit mAbs and one rabbit polyclonal antibody to the SARS-CoV-2 S-RBD were developed as described in the Methods section. Thirty-three mouse and three rabbit hybridomas were selected in the primary hybridoma screens, cloned, and further analyzed by ELISA for reactivities using the S-RBD protein, and the results are summarized in Table 1. A direct ELISA using purified S-RBD attached to the ELISA plate wells at alkaline pH was used as the primary screening method to detect hybridomas positive for antibodies to the S-RBD antigen. Subsequently, the binding activities were tested following denaturation and reduction of the S-RBD antigen as described in the Methods section. As indicated in Table 1, only 2/33 mouse mAbs (S11.A and S21.D) and 1/3 rabbit mAbs retained activity, indicating that most of these mAbs recognized some conformational components of the S-RBD antigen. All of the mouse and rabbit mAbs, except for S3.E and S24.H (IgM), were IgG isotypes.

Several mAbs were used to capture the S-RBD antigen in a sandwich ELISA to determine whether there were any mAbs that showed competitive binding to the S-RBD. One mouse mAb (S3.E IgM) and the three rabbit mAbs (#14, #24, #77) were selected as primary capture mAbs and the remaining mouse mAbs were scanned for reactivity to the captured S-RBD antigen. Lack of signals from the detection mAb was interpreted to indicate that the binding mAb recognized a similar or adjacent epitope on the S-RBD. The data in Table 1 (and Supplemental Figures S1–S3) indicated that 17/32 mouse mAbs were unable to bind to the S3.E-captured S-RBD. Similarly, 22/33 and 18/33 mouse mAbs were blocked from binding to the S-RBD captured by rabbit mAbs #14 and #77, respectively. Notably, the mouse mAbs that failed to bind to the captured S-RBD were essentially the same for the capture ELISA using S3.E, #14, and #77, suggesting that these three mAbs bound to either the same or adjacent epitope on the S-RBD protein fragment. In contrast, only 2/33 mouse mAbs were blocked from binding to S-RBD captured by rabbit mAb #24. Notably, these two mouse mAbs (S11.A and S21.D) were the only two mouse mAbs that recognized denatured and reduced S-RBD antigen in a direct ELISA (Table 1). It is also interesting to note that mouse mAb S3.E and rabbit mAb #14 were SARS-CoV neutralizing mAbs, whereas rabbit mAbs #24 and #77 were non-neutralizing (Table 1 and Table 2).

**Figure 1:**
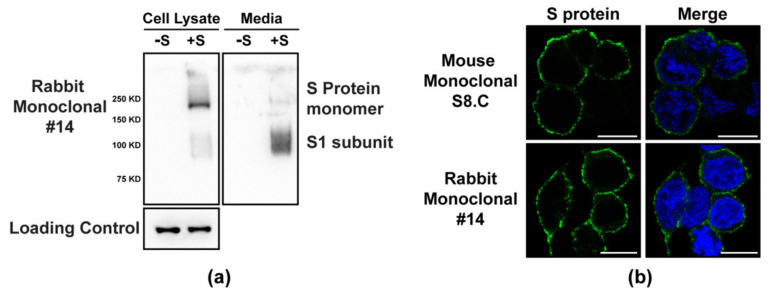
(**a**) Cell lysates and supernatants were collected from 293 cells transfected with constructs expressing S (+S) or N (–S) proteins of SARS-CoV-2 and subjected to Western blot analysis. Blots were probed with rabbit monoclonal #14 or actin (loading control for cell lysate only). (**b**) 293T cells transfected with a plasmid expressing SARS-CoV-2 S protein and subjected to IF analysis with S8.C mouse mAb or S #14 rabbit mAb at 24 h post-transfection.

We tested various S-RBD mAbs for the ability to detect S protein in common immune-assays. We identified several antibodies that bound strongly to the S protein in Western blot analyses. Only those mAbs that showed strong signals in Westerns were identified as positive. We also observed that some of the mAbs showed weak signals, and these were considered as negative in Table 1, although these latter mAbs may show positivity with higher antibody concentrations. Rabbit mAb #14 showed excellent binding in Western blots (Figure 1a). Following transfection of 293T cells, S protein could be detected in both cell lysates and the extracellular media. The size of the predominant form of cell-associated S protein correlated with the full-length monomer, while a shorter S protein form, most likely representing the cleaved form of S protein, predominated in the media. A loading control is included to show equal loading of the cell lysates (Figure 1a) and the two supernatant samples were processed in a similar manner to each other as described in the Material and Methods. We observed that the binding to S protein in Western blots by both rabbit polyclonal and mouse and rabbit monoclonal antibodies was best achieved using denatured, non-reducing gel conditions. Standard denaturing and reducing Western blot conditions showed poor reactivity to S protein. We also tested several of the antibodies for the ability to detect S protein in an immunofluorescence (IF) assay. Several antibodies detected S protein at the plasma membrane in transfected cells, consistent with its localization in the absence of other viral proteins (Figure 1b). The absence of staining in un-transfected cells clearly indicated the specificity of the antibody (Supplemental Figure S4). Thus, the panel of S protein antibodies has utility in addressing questions related to basic coronavirus research.

We next tested whether the panels of S-RBD-reactive mAbs and rabbit polyclonal sera could neutralize native SARS-CoV-2 using both standard cell culture supernatants (antibody ½ max titers ranged from 500–2000) and selected high-titer antibody from hybridomas adapted to serum-free media and grown in CELLine cell culture devices (antibody ½ max titers ranged between 800–20,000) (Table 2). Six mouse and one rabbit mAb demonstrated neutralizing activity, as did the rabbit polyclonal anti-S-RBD sera. The most potent neutralizer in the mAb set was S3.E (IgM) as determined by a comparison between ELISA and neutralization titers (ratio ranging between 1–8). The remaining neutralizing mAbs were all IgG isotype and showed less potent neutralizing activity (ratio ranging between 50–200) but showed stronger neutralizing activity when higher titer antibodies were prepared from serum-free adapted hybridoma cultures (Table 2). Monoclonal antibodies targeting surface conformational epitopes are more likely to have functional importance and neutralizing capacity. Conformational surface epitopes were the major source of sites targeted by neutralizing antibodies against HPVs [21]. Accordingly, the three S protein mAbs (S11.A, S21.D, and #24) that recognized denatured/linear epitopes were non-neutralizers.

We also noted that the rabbit hybridomas, in general, produced lower titers of antibody when grown in standard culture conditions as compared with mouse hybridomas, but could produce high-titer antibodies following adaptation to serum-free culture conditions (Table 2). Thus, the panel of S protein antibodies has the potential to be developed as clinical reagents in antagonizing COVID-19.

### 3.2. N Protein Antibodies Bind throughout the N Protein and Vary in Their Coronavirus Specificities

A set of mouse and rabbit monoclonal antibodies to the SARS-CoV-2 N protein were developed as described in the Methods section. Sixty mouse and one rabbit hybridoma antibodies were selected in the primary hybridoma screen, cloned, and further analyzed by ELISA for reactivities using the N protein from SARS-CoV, SARS-CoV-2, and MERS-CoV. The results are summarized in Table 2. A direct ELISA using the SARS-CoV-2 N protein attached to the ELISA plate wells at alkaline pH was used as the primary screening method to detect hybridomas positive for antibody to the N protein. Next, we tested the binding activities of the antibodies from the hybridomas following denaturation and reduction of the N protein antigen. We observed that only 3/60 mouse mAbs lost reactivity indicating that most of these mAbs recognized linear epitopes of the N protein. Monoclonal antibodies that target surface conformational epitopes are more likely to have both higher levels of specificity and more often functional importance, while buried linear epitopes are excellent for detection in Western blots where high levels of denaturation expose these internal sites. Interestingly, all three N-reactive mAbs (N2.20A, N4.5D, and N10.13C) that recognized a conformational epitope were SARS-CoV-2 type-specific. It was not an exclusive requirement, as there were a couple of others (N1.15C, N8.3B, N10.15H, and N10.29B) that were also type-specific but recognized denatured antigen. The remainder were all able to recognize linear epitopes and were mostly cross-reactive.

**Figure 2:**
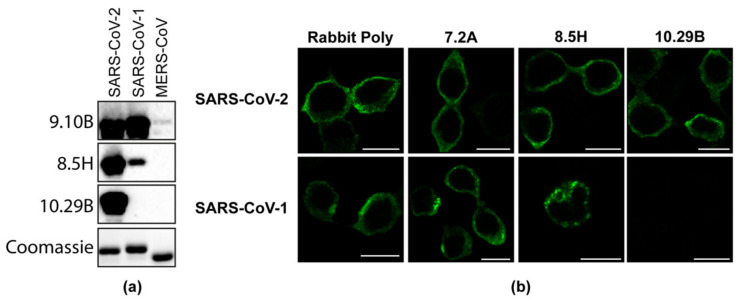
(**a**) Western blot showing purified N proteins from SARS-CoV, SARS-CoV-2, and MERS-CoV probed with monoclonal N antibodies 9.10B, 8.5H, and 10.29B. A Coomassie-stained polyacrylamide gel shows the equal loading of the proteins. (**b**) 293T cells transfected with a plasmid expressing SARS-CoV-2 or the SARS-CoV N protein were fixed at 24 h post-transfection and probed with the rabbit polyclonal serum, or 7.2A, 8.5H, and 10.29B mouse monoclonal antibodies.

We next tested these mAbs for cross-reactivity to the N protein of related coronaviruses and found that 7/60 mouse mAbs were uniquely reactive to the SARS-CoV-2 N protein, 20/60 mAbs were reactive to SARS-CoV-2 and the SARS-CoV N protein, and 33/60 showed cross-reactivity to SARS-CoV-2, SARS-CoV, and MERS-COV N proteins (Table 3). One rabbit mAb (#54) showed reactivity to both SARS-CoV-2 and the SARS-CoV N protein and bound to denatured, reduced N protein in a direct ELISA (Table 3). Representative antibodies from each category were selected for Western blot and IF analysis (Figure 2a,b), confirming both the specificity (Supplemental Figure S4) and the distinct detection properties of these antibodies for different coronavirus N proteins.

To assess whether there was competition between the rabbit mAb and the mouse mAbs for binding to the same or adjacent epitope on the N protein, we tested rabbit mAb (#54) in a capture ELISA for the SARS-CoV-2 N protein. The ELISA assay demonstrated that only three mouse mAbs failed to bind the captured N protein (mAbs N2.20A, N4.5D, and N10.13C, Table 3). Interestingly, these were the only three mouse mAbs that showed SARS-CoV-2-specific reactivity to the N protein and lost ELISA-binding activity when the N protein antigen was denatured and reduced (Table 3). Rabbit mAb #54, in contrast, was bound to denatured and reduced N protein and was cross-reactive to SARS-CoV N protein (Table 3).

**Figure 3:**
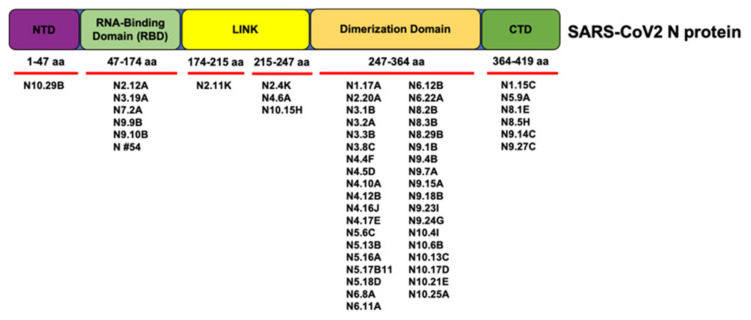
Schematic depicting the regions of the N protein targeted by each antibody based on competitive ELISA results.

We used a series of expressed truncations to map the location of the epitopes recognized by the panel of anti-N protein mAbs (Table 3 and Figure 3). Most of the mouse mAbs (42/60) mapped to an internal region of the N protein (aa 247–364); one mAb mapped to aa 1–47; 7/60 mAbs mapped to aa 47–174; one mAb mapped to aa 174–215; 3/60 mAbs mapped to aa 215–247, and 6/60 mAbs mapped to aa 364–419 (Table 3). The seven SARS-CoV-2 type-specific mAbs recognized several different epitopes over the entire protein; the rabbit mAb #54 recognized aa 47–174 (Table 3). Figure 3 summarizes the regions bound by the different N protein antibodies.

## 4. Discussion

Despite encouraging trends in the global effort against SARS-CoV-2, variants of concern and regions with low levels of collective immunity threaten to undermine this progress, as has been demonstrated by the rise of the delta and lambda variants. Thus, there is a need to continue studying the antigenic and structural properties of SARS-CoV-2, particularly with respect to the interaction between the virus and the host immune system.

The goals of this study were to develop panels of mouse and rabbit monoclonal antibodies to SARS-CoV-2 S and N proteins and test these reagents in various immunoassays for function (neutralizing activity) and specificity. Since both the S and N proteins are the major targets of the antibody response following infection with SARS-CoV or SARS-CoV-2 [1,2,3], this panel will provide key reagents in furthering our understanding of the role of these proteins during the replication cycle of the virus as well as in understanding how these key proteins are recognized by the immune system.

It is important to have antigen detection assays as they can ensure early detection of infection. The high level of amino acid conservation amongst the coronavirus N proteins [22] prevent simple virus detection methods using polyclonal anti-N protein antisera and sera from patients who have cleared the infection, given the commonality of coronavirus infections from various related family members. With respect to specificity, the majority of the generated mAbs to the SARS-CoV-2 N protein showed cross-reactivity to several related coronavirus N proteins (SARS-CoV and MERS-CoV), but there were several that also showed unique reactivity to only the SARS-CoV-2 N protein. This is useful in virus detection assays of clinical samples. The combination of mouse and rabbit mAbs to the N protein in which various mAbs show a lack of competitive binding to the N protein would enable capture/detection assays to be developed to classify the SARS-CoV-2 N protein in clinical samples in which other co-incident coronavirus types may be present. Antibodies have been used to generate assays previously for HPV [23]; therefore, these antibodies will be extremely useful in developing diagnostic tools that can distinguish between various coronavirus infections. Furthermore, future work with these mAb panels can determine whether discrimination of N and/or S proteins are suitable for distinguishing different SARS-CoV-2 variants of concern.

The panel of N and S antibodies both show utility in several immunodetection assays. The combination of mouse and rabbit mAbs to SARS-CoV-2 S proteins (targeted to the RBD) could be used to set up capture/detection assays focused on S proteins of various coronaviruses. At present, the ability of these panels of reagents to discriminate between related and/or variants of S proteins awaits further testing. The current threat with the upcoming variants of concern and the efficacy of the vaccines against these variants is of importance. These panels of antibodies may be used to study how these variants behave differently and what provides them the additional fitness to be highly transmissible compared to the wild-type variant.

Continued research on the basic replication processes of SARS-CoV-2 and other coronaviruses is paramount to overcoming the current pandemic, as well as in dealing with future crossover events. For example, the SARS-CoV-2 N protein is an abundant RNA-binding protein critical for viral genome packaging, yet the molecular details that underlie this process are poorly understood [24]. Our panel of antibodies recognizes specific domains across the length of the N protein, with six of them specific to the RNA-binding domain. We believe these antibodies can facilitate in studying the competitive binding and specific RNA-binding studies which will be critical in understanding viral genome packing. Thus, we have developed a panel of antibodies with potential utility in both basic research and the treatment and diagnosis of SARS-CoV-2.

## Figures and Tables

**Table 1:** Reactivities of anti-S-RBD monoclonal antibodies.

	ELISA ^a^ Signals to Various Antigens and Competitive ELISA							
Mab (Mouse)	CoV-2 S-RBD (intact)	CoV-2 S-RBD (D and R)	Comp with S3.E ^b^	Comp with #24 ^c^	Comp with #14 ^d^	Comp with #77 ^e^	N (Native)	Isotype
S1.D	+	−	−	−	−	−	−	IgG1
S2.D	+	−	+	−	+	+	−	IgG1
S3.E	+	−	N/A	−	+	+	+	IgM
S5.F	+	−	+	−	−	+	−	IgG1
S7.A	+	−	+	−	+	+	−	IgG1
S8.C	+	−	+	−	+	+	+	IgG2b
S11.A	+	+	−	+	−	−	−	IgG2b
S15.B	+	−	+	−	+	+	−	IgG1
S16.C	+	−	+	−	+	+	−	IgG1
S18.C	+	−	−	−	−	−	−	IgG1
S19.K	+	−	−	−	−	−	−	IgG1
S20.I	+	−	+	−	+	+	−	IgG1
S21.D	+	+	−	+	−	−	−	IgG1
S22.D	+	−	+	−	+	+	−	IgG1
S24.H	+	−	+	−	−	−	−	IgM
S27.D	+	−	+	−	+	+	+	IgG1
S28.F	+	−	+	−	+	+	+	IgG1
S30.B	+	−	−	−	+	−	−	IgG1
S33.B	+	−	+	−	+	+	+	IgG1
S34.G	+	−	+	−	+	+	−	IgG2b
S35.I	+	−	+	−	+	+	−	IgG1
S36.E	+	−	−	−	−	−	−	IgG1
S37.E	+	−	−	−	−	−	−	IgG1
S38.C	+	−	−	−	+	+	−	IgG1
S39.B	+	−	−	−	−	−	−	IgG1
S40.B	+	−	−	−	+	−	−	IgG1
S43.E	+	−	+	−	+	+	+	IgG1
S45.A	+	−	−	−	+	−	−	IgG1
S46.C	+	−	−	−	+	−	−	IgG1
S48.E	+	−	−	−	+	−	−	IgG1
S49.C	+	−	−	−	−	−	−	IgG1
S52.C	+	−	+	−	+	+	−	IgG2b
S53.B	+	−	+	−	+	+	−	IgG1
Mab (Rabbit)	CoV-2 S-RBD (intact)	CoV-2 S-RBD (D and R)	Comp with S3.E ^b^	Comp with S8.C ^f^	Comp with S11.A ^g^	Comp with S43.E ^h^	Neut (Native)	Isotype
S #14	+	−	+	+	−	+	+	IgG
S #24	+	+	−	−	+	−	−	IgG
S #77	+	−	+	+	−	+	−	IgG

^a^ ELISA values are considered positive (+) when the O.D. ranges from 0.1 to 2.5 and negative (–) when the O.D. is <0.1. ^b^ Mouse monoclonal S3.E was used to capture the S-RBD antigen. ^c^ Rabbit monoclonal #24 was used to capture the S-RBD antigen. ^d^ Rabbit monoclonal #14 was used to capture the S-RBD antigen. ^e^ Rabbit monoclonal #77 was used to capture the S-RBD antigen. ^f^ Mouse monoclonal S8.C was used to capture the S-RBD antigen. ^g^ Mouse monoclonal S11.A used to capture the S-RBD antigen. ^h^ Mouse monoclonal S43.E was used to capture the S-RBD antigen. NT: (Not tested); N/A: (Not applicable); D: Denatured; R: Reduced; N: Neutralizing.

**Table 2:** Neutralizing activity of mAbs and pAbs.

Antibody Designation ^a^	Origin and Isotype	ELISA Titer ^b^	Neutralizing Titer ^c^	Ratio ^d^
S3.E (cell culture s/n ^e^)	Mouse Mab (IgM)	800	640	1.25
S3.E (CELLine s/n)	Mouse Mab (IgM)	10,000	1280	7.8
S8.C (cell culture s/n)	Mouse Mab (IgG2b)	1000	10	100
S8.C (CELLine s/n)	Mouse Mab (IgG2b)	20,000	320	62.5
S27.D (cell culture s/n)	Mouse Mab (IgG1)	2000	10	200
S28.F (cell culture s/n)	Mouse Mab (IgG1)	2000	10	200
S33.B (cell culture s/n)	Mouse Mab (IgG1)	2000	10	200
S43.E (cell culture s/n)	Mouse Mab (IgG1)	2000	40	50
S #14 (CELLine s/n)	Rabbit Mab (IgG)	1000	10	100
S #24 (CELLine s/n)	Rabbit Mab (IgG)	10,000	0	N/A
S #77 (CELLine s/n)	Rabbit Mab (IgG)	800	0	N/A
S 5393 (Rabbit serum)	Rabbit Pab	500	80	6.25
N 5394 (Rabbit serum)	Rabbit Pab (anti-N)	<10	0	N/A

^a^ Monoclonal designation number and rabbit number for the source of antibody. ^b^ ELISA titer is determined as dilution of media/serum that represents ½ maximum O.D. binding. ^c^ Neutralization titer determined as described in the Methods section. ^d^ Ratio is the ELISA titer divided by the Neutralization titer. ^e^ supernatant. N/A: Not applicable.

**Table 3:** mAbs against CoV-2 N protein.

	ELISA ^a^ Signals for Various Antigens						
Mab Clone (Mouse)	CoV-2 ^b^ (intact)	CoV-2 ^c^ (D and R)	CoV ^d^ (Intact)	MERS ^e^ (Intact)	Region (aa) ^f^	Western ^g^ (CoV-2)	Isotype
N1.15C	+	+	−	−	364–419	+	IgG1
N1.17A	+	+	+	+	247–364	+	IgG2b
N2.4K	+	+	+	+	215–247	+	IgG1
N2.11K	+	+	+	+	174–215	+	IgG1
N2.12A	+	+	+	+	47–174	−	IgG1
N2.20A	+	−	−	−	247–364	+	IgG1
N3.1B	+	+	+	+	247–364	−	IgG1
N3.2A	+	+	+	+	247–364	+	IgG1
N3.3B	+	+	+	+	247–364	+	IgG2b
N3.8C	+	+	+	+	247–364	+	IgG1
N3.19A	+	+	+	+	47–174	+	IgG1
N4.4F	+	+	+	+	247–364	−	IgG1
N4.5D	+	−	−	−	247–364	−	IgG1
N4.6A	+	+	+	+	215–247	+	IgM
N4.10A	+	+	+	+	247–364	+	IgG1
N4.12B	+	+	+	−	247–364	−	IgG1
N4.16J	+	+	+	+	247–364	+	IgG1
N4.17E	+	+	+	−	247–364	−	IgG1
N5.6C	+	+	+	+	247–364	+	IgG1
N5.9A	+	+	+	−	364–419	−	IgG1
N5.13B	+	+	+	+	247–364	−	IgG1
N5.16A	+	+	+	−	247–364	−	IgG1
N5.17B11	+	+	+	−	247–364	−	IgG1
N5.18D	+	+	+	−	247–364	−	IgG1
N6.8A	+	+	+	+	247–364	−	IgG1
N6.11A	+	+	+	+	247–364	−	IgG1
N6.12B	+	+	+	−	247–364	−	IgG1
N6.22A	+	+	+	−	247–364	−	IgG1
N7.2A	+	+	+	+	47–174	+	IgG1
N7.28C	+	+	+	+	247–364	+	IgG2b
N7.17B	+	+	+	−	247–364	−	IgG1
N7.19A	+	+	+	−	47–174	−	IgG1
N7.22D	+	+	+	−	247–364	−	IgG2a
N7.26A	+	+	+	+	247–364	+	IgG2b
N8.1E	+	+	+	+	364–419	−	IgG2b
N8.2B	+	+	+	−	247–364	−	IgG1
N8.3B	+	+	−	−	247–364	+	IgG2b
N8.5H	+	+	+	−	364–419	+	IgG1
N8.8E	+	+	+	+	47–174	+	IgG1
N8.29B	+	+	+	−	247–364	−	IgG1
N9.1B	+	+	+	+	247–364	−	IgG1
N9.4B	+	+	+	+	247–364	−	IgG1
N9.7A	+	+	+	−	247–364	−	IgG1
N9.9B	+	+	+	−	47–174	−	IgG1
N9.10B	+	+	+	+	47–174	−	IgG2b
N9.13A	+	+	+	+	247–364	−	IgG1
N9.14C	+	+	+	+	364–419	−	IgG1
N9.15A	+	+	+	+	247–364	−	IgG1
N9.18B	+	+	+	−	247–364	−	IgG1
N9.23I	+	+	+	−	247–364	−	IgG1
N9.24G	+	+	+	+	247–364	−	IgG1
N9.27C	+	+	+	−	364–419	−	IgG1
N10.4I	+	+	+	−	247–364	−	IgG1
N10.6B	+	+	+	+	247–364	−	IgG1
N10.13C	+	−	−	−	247–364	−	IgG1
N10.15H	+	+	−	+	215–247	−	IgG1
N10.17D	+	+	+	+	247–364	−	IgG1
N10.21E	+	+	+	+	247–364	−	IgG1
N10.25A	+	+	+	+	247–364	−	IgG1
N10.29B	+	+	−	−	1–47	−	IgG1
Mab clone (Rabbit)	CoV-2 ^b^ (intact)	CoV-2 ^c^(D and R)	CoV ^d^ (intact)	MERS ^e^ (intact)	Region (aa) ^f^	Western ^g^ (CoV-2)	Isotype
N #54	+	+	+	−	47–174	NT	IgG

^a^ ELISA values are considered positive (+) when the O.D. ranges from 0.1 to 2.5 and negative (–) when the O.D. is <0.1. ^b^ SARS-CoV-2 N protein antigen attached with alkaline buffer, pH 9.6. ^c^ SARS-CoV-2 N protein antigen attached with alkaline buffer after boiling in buffer with 2ME. ^d^ SARS-CoV N protein antigen attached with alkaline buffer, pH 9.6. ^e^ MERS-CoV N protein antigen attached with alkaline buffer, pH 9.6. ^f^ SARS-CoV-2 N protein amino acid ranges that yield a positive ELISA signal. ^g^ Western reactivity to SARS-CoV-2 showing strong signals. D: Denatured; R: Reduced; CoV: SARS-CoV; CoV-2: SARS-CoV-2; MERS: MERS-CoV.

## Data Availability

The data presented in this study are available on request from the corresponding author.

## Supplementary Materials

The following are available online at https://www.mdpi.com/article/10.3390/v13101899/s1, Figure S1: Capture ELISA using mouse mAb S3.E (IgM isotype), Figure S2. Capture ELISA using 3 rabbit anti-S-RBD mAbs, Figure S3. Capture ELISA using select mouse mAbs, Figure S4. Immunofluorescence of untransfected cells to show specificity of the N and S antibodies to their respective antigens.
