# Supplementary material for: Monoclonal Antibodies to S and N SARS-CoV-2 Proteins as Probes to Assess Structural and Antigenic Properties of Coronaviruses
Source: Viruses. 2021 Sep 22;13(10):1899. doi: 10.3390/v13101899 (PMC8537396; doi:10.3390/v13101899)

Supplemental Figure S1. Capture ELISA using mouse mAb S3.E (IgM isotype); detection using mouse mAbs. A sandwich ELISA was prepared by capturing mouse mAb S3.E using a goat-anti mouse IgM polyclonal antibody. S-RBD protein was captured by the mouse mAb S3.E, then the set of mouse mAbs to S-RBD (except S3.E) were added followed by detection secondary anti-mouse antibody (anti-IgG1, anti-IgG2a, anti-IgG2b) as determined by the detecting mAb isotype.

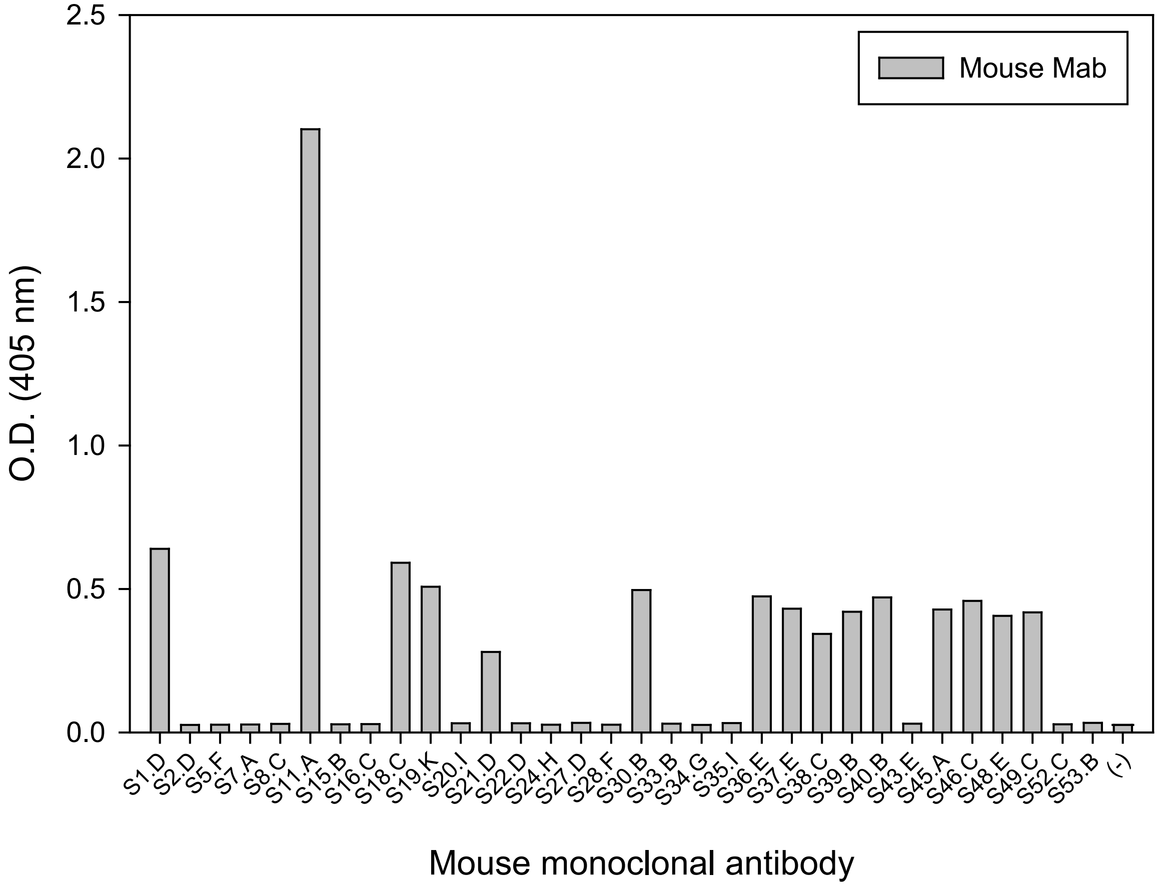

Supplemental Figure S2. Capture ELISA using 3 rabbit anti-S-RBD mAbs; detection using mouse mAbs. A sandwich ELISA was prepared by capturing 3 rabbit mAbs (mAbs #14, #24 and #77) using a goat-anti rabbit IgG polyclonal antibody. S-RBD protein was captured by the rabbit mAbs, then the set of mouse mAbs to S-RBD were added followed by detection with a secondary goat anti-mouse antibody (anti-IgG and anti-IgM).

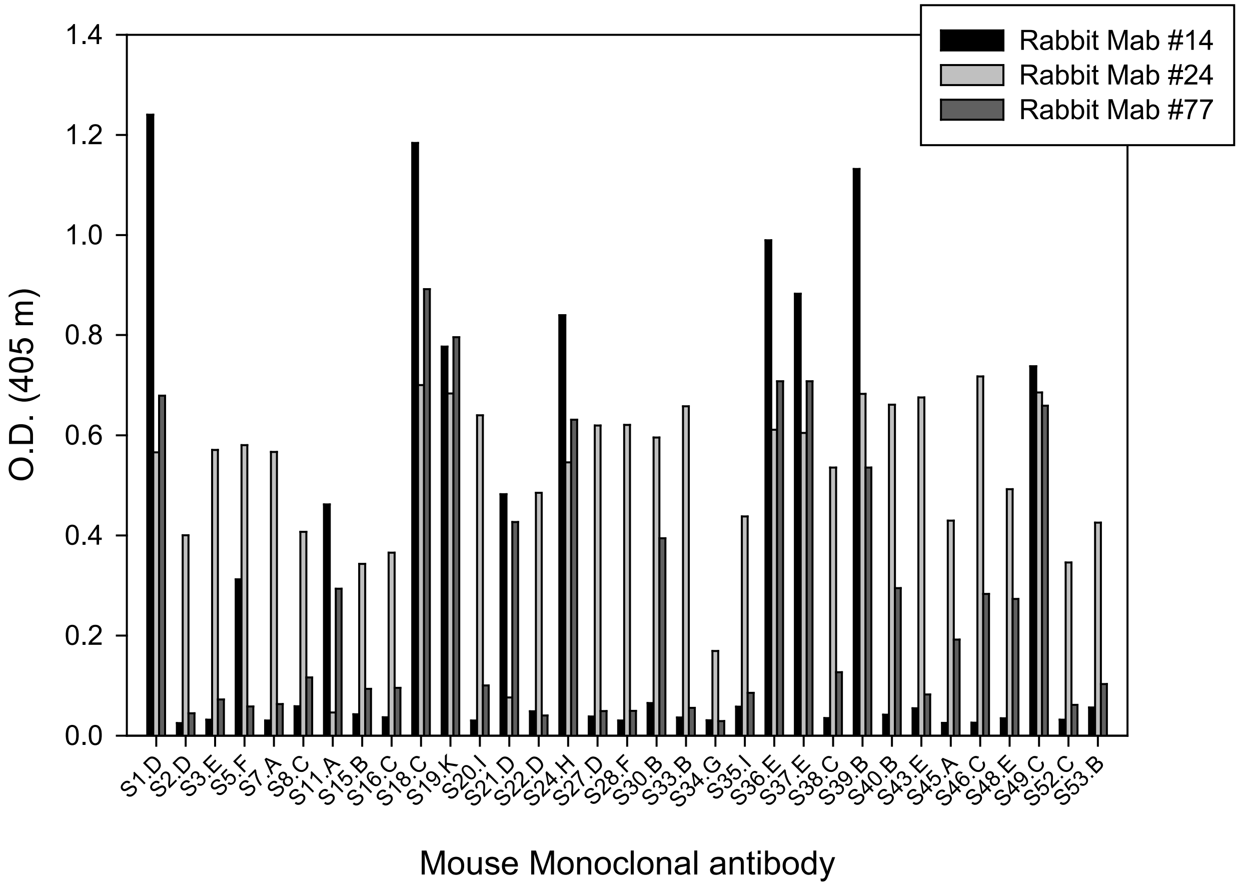

Supplemental Figure S3. Capture ELISA using select mouse mAbs; detection using rabbit mAbs. A sandwich ELISA was prepared by capturing selected mouse mAbs (S3.E, S8.C, S11.A, S18.C and S43.E) using isotype-specific goat-anti mouse polyclonal antibody. S-RBD protein was captured by the mouse mAbs, then three rabbit mAbs (#14, #24 and #77) to S-RBD were added followed by detection using a secondary anti-rabbit antibody.

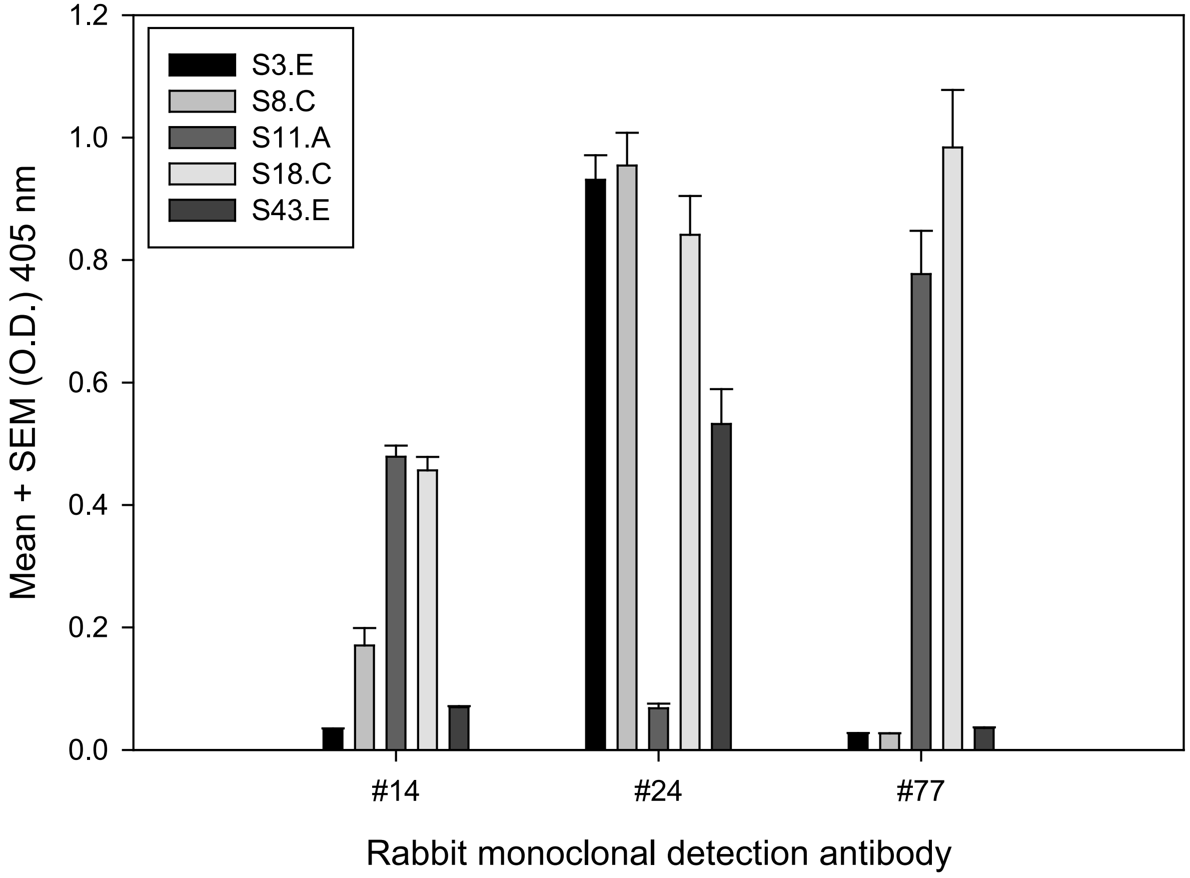

Supplemental Figure S4. Immunofluorescence of untransfected cells to show specificity of the N and S antibodies to their respective antigens. * denotes untransfected cell next to stained transfected cell expressing S protein

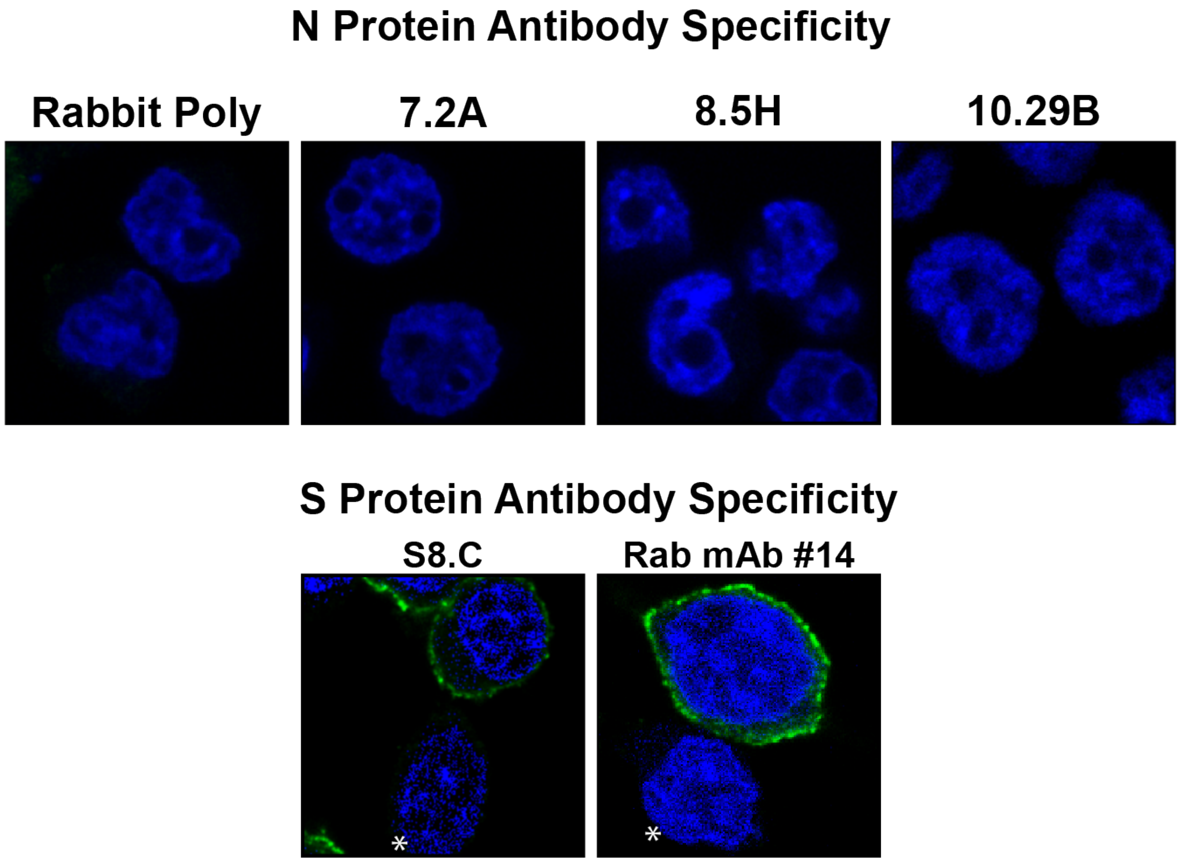

Supplement: Supplementary file 1 [file viruses-13-01899-s001.zip › viruses-1367584-supplementary.pdf]
